# Supplementary material for: Training set augmentation and biology-aware harmonization improve radiomic models for lung cancer prediction in indeterminate nodules
Source: Sci Rep. 2026 Jun 20;16:28296. doi: 10.1038/s41598-026-57670-4 (PMC13558760; doi:10.1038/s41598-026-57670-4)
Supplement: Supplementary file 1 [file 41598_2026_57670_MOESM1_ESM.docx]

**Figure S1. Scan classification for three representative PNs.** In A and B, endpoint diagnosis was confirmed using another diagnostic method after all scans were acquired. A) A representative PN with some scans classified into the early-development dataset and some scans classified into the augmentation malignant dataset. B) A representative PN with some scans classified into the early-development dataset and some scans classified into the augmentation benign dataset. C) A representative PN with all scans classified into the early-development dataset (unconfirmed benign in Fig. 2).

**Table S1.** **Instances of the acquisition parameters of interest in the three datasets.** Acquisition parameters which were controlled for in this study are placed at the top of this table, and those not controlled for in this study are placed below the divider.

| **Parameter** | **Protocol** | **Early-Development**  **Occurrence**  **(# Scans)** | **Augmentation Malignant Occurrence**  **(# Scans)** | **Augmentation**  **Benign Occurrence**  **(# Scans)** |
| --- | --- | --- | --- | --- |
| CE | Non-CE | 80 | 57 | 18 |
|  | CE | 26 | 132 | 18 |
| Focal Spots | 1.2 | 25 | 90 | 18 |
|  | 0.7 | 54 | 81 | 10 |
|  | 0.8 | 8 | 11 | 7 |
|  | 0.9 | 19 | 7 | 1 |
| KVP | 120 | 98 | 171 | 30 |
|  | 100 | 8 | 17 | 6 |
| Scanner  Manufacturer | SIEMENS | 39 | 28 | 22 |
|  | GE MEDICAL SYSTEMS | 67 | 161 | 14 |
| Scanner Model Name | SOMATOM Definition AS | 25 | 13 | 8 |
|  | SOMATOM Force | 8 | 11 | 7 |
|  | Revolution HD | 6 | 15 | 1 |
|  | LightSpeed VCT | 31 | 51 | 5 |
|  | Discovery CT750 HD | 30 | 75 | 8 |
|  | SOMATOM Definition Flash | 6 | 3 | 1 |
|  | Sensation 64 | 0 | 0 | 6 |
| Convolution Kernel | 0 | 0 | 0 | 6 |
|  | BODY FILTER | 67 | 161 | 14 |
|  | FLAT | 33 | 25 | 15 |
|  | WEDGE_2 | 2 | 0 | 1 |
|  | ['Br59f', '2'] | 19 | 6 | 1 |
|  | ['I30f', '2'] | 1 | 6 | 1 |
|  | LUNG | 54 | 39 | 14 |
|  | ['I70f', '2'] | 7 | 2 | 5 |
|  | I70f | 0 | 0 | 1 |
|  | ['Bl64d', '2'] | 8 | 9 | 7 |
|  | ['I70f', '3'] | 1 | 0 | 1 |
|  | ['I30f', '3'] | 1 | 0 | 1 |
|  | B60f | 0 | 0 | 5 |
| Data Collection Diameter | 500 | 106 | 189 | 36 |
| Slice Thickness | 1.25 | 67 | 161 | 14 |
|  | 3 | 0 | 0 | 5 |
|  | 5 | 0 | 0 | 1 |
|  | 1.5 | 37 | 28 | 16 |

**Table S2.** **Frequency of net acquisition dependence for radiomic features** **in training sets with 100% augmentation.** Frequencies are calculated over all features and trials (n = 107 features × 50 trials = 5350).

|  | **Collective Harmonization (%)** | | **Covariate Harmonization (%)** | | **Separate Harmonization (%)** | |
| --- | --- | --- | --- | --- | --- | --- |
|  | **Augmentation Malignant** | **Augmentation Benign** | **Augmentation Malignant** | **Augmentation Benign** | **Augmentation Malignant** | **Augmentation Benign** |
| Shape Elongation | 0 | 0 | 0 | 0 | 0 | 0 |
| Shape Flatness | 0 | 0 | 0 | 0 | 0 | 0 |
| Shape Least Axis Length | 100 | 100 | 0 | 0 | 0 | 0 |
| Shape Major Axis Length | 98 | 100 | 8 | 44 | 0 | 0 |
| Shape Maximum 2D Diameter Column | 100 | 100 | 2 | 34 | 0 | 0 |
| Shape Maximum 2D Diameter Row | 98 | 100 | 4 | 46 | 0 | 0 |
| Shape Maximum 2D Diameter Slice | 100 | 100 | 0 | 38 | 0 | 0 |
| Shape Maximum 3D Diameter | 100 | 100 | 4 | 44 | 0 | 0 |
| Shape Mesh Volume | 100 | 100 | 100 | 46 | 0 | 0 |
| Shape Minor Axis Length | 100 | 100 | 0 | 36 | 0 | 0 |
| Shape Sphericity | 100 | 72 | 0 | 2 | 0 | 0 |
| Shape Surface Area | 100 | 100 | 48 | 94 | 0 | 0 |
| Shape Surface Volume Ratio | 98 | 36 | 0 | 0 | 0 | 0 |
| Shape Voxel Volume | 100 | 100 | 100 | 46 | 0 | 0 |
| First Order 10 Percentile | 100 | 0 | 0 | 0 | 0 | 0 |
| First Order 90 Percentile | 0 | 30 | 4 | 58 | 0 | 0 |
| First Order Energy | 100 | 100 | 46 | 86 | 0 | 0 |
| First Order Entropy | 90 | 6 | 78 | 48 | 0 | 0 |
| First Order Interquartile Range | 54 | 0 | 0 | 0 | 0 | 0 |
| First Order Kurtosis | 22 | 0 | 24 | 0 | 0 | 0 |
| First Order Maximum | 2 | 0 | 2 | 10 | 0 | 0 |
| First Order Mean | 52 | 28 | 2 | 56 | 0 | 0 |
| First Order Mean Absolute Deviation | 90 | 0 | 14 | 0 | 0 | 0 |
| First Order Median | 92 | 0 | 12 | 0 | 0 | 0 |
| First Order Minimum | 100 | 100 | 98 | 10 | 100 | 0 |
| First Order Range | 10 | 0 | 2 | 4 | 0 | 0 |
| First Order Robust Mean Absolute Deviation | 48 | 8 | 0 | 0 | 0 | 0 |
| First Order Root Mean Squared | 100 | 0 | 16 | 0 | 0 | 0 |
| First Order Skewness | 98 | 8 | 0 | 0 | 0 | 0 |
| First Order Total Energy | 100 | 100 | 46 | 86 | 0 | 0 |
| First Order Uniformity | 56 | 6 | 76 | 56 | 0 | 0 |
| First Order Variance | 36 | 74 | 6 | 80 | 0 | 0 |
| GLCM Autocorrelation | 88 | 0 | 6 | 0 | 0 | 0 |
| GLCM Cluster Prominence | 4 | 16 | 0 | 52 | 0 | 0 |
| GLCM Cluster Shade | 28 | 4 | 2 | 0 | 0 | 0 |
| GLCM Cluster Tendency | 40 | 18 | 0 | 36 | 0 | 0 |
| GLCM Contrast | 52 | 56 | 48 | 58 | 0 | 0 |
| GLCM Correlation | 0 | 100 | 72 | 12 | 0 | 0 |
| GLCM Difference Average | 62 | 44 | 28 | 46 | 0 | 0 |
| GLCM Difference Entropy | 98 | 8 | 84 | 56 | 0 | 0 |
| GLCM Difference Variance | 0 | 48 | 28 | 72 | 0 | 0 |
| GLCM Inverse Difference | 72 | 98 | 10 | 58 | 0 | 0 |
| GLCM Inverse Difference Moment | 72 | 100 | 36 | 94 | 0 | 0 |
| GLCM Inverse Difference Moment Normalized | 98 | 26 | 0 | 22 | 0 | 0 |
| GLCM Inverse Difference Normalized | 98 | 26 | 0 | 28 | 0 | 0 |
| GLCM Informational Measure of Correlation 1 | 100 | 24 | 92 | 0 | 0 | 0 |
| GLCM Informational Measure of Correlation 2 | 98 | 0 | 0 | 0 | 0 | 0 |
| GLCM Inverse Variance | 74 | 86 | 2 | 72 | 0 | 0 |
| GLCM Joint Average | 80 | 0 | 14 | 0 | 0 | 0 |
| GLCM Joint Energy | 100 | 8 | 46 | 60 | 0 | 0 |
| GLCM Joint Entropy | 100 | 44 | 98 | 0 | 0 | 0 |
| GLCM Maximum Correlation Correlation | 100 | 0 | 100 | 0 | 0 | 0 |
| GLCM Maximum Probability | 88 | 52 | 46 | 82 | 0 | 0 |
| GLCM Sum Average | 80 | 0 | 14 | 0 | 0 | 0 |
| GLCM Sum Entropy | 96 | 88 | 22 | 0 | 0 | 0 |
| GLCM Sum Squares | 70 | 30 | 26 | 54 | 0 | 0 |
| GLDM Dependence Entropy | 100 | 100 | 0 | 32 | 0 | 0 |
| GLDM Dependence Non-uniformity | 100 | 98 | 4 | 0 | 0 | 0 |
| GLDM Dependence Non-uniformity Normalized | 98 | 80 | 0 | 34 | 0 | 0 |
| GLDM Dependence Variance | 100 | 100 | 100 | 100 | 0 | 0 |
| GLDM Gray Level Non-uniformity | 100 | 98 | 100 | 62 | 100 | 0 |
| GLDM Gray Level Variance | 36 | 74 | 6 | 80 | 0 | 0 |
| GLDM High Gray Level Emphasis | 80 | 0 | 8 | 0 | 0 | 0 |
| GLDM Large Dependence Emphasis | 100 | 100 | 100 | 100 | 0 | 0 |
| GLDM Large Dependence High Gray Level Emphasis | 96 | 100 | 100 | 100 | 0 | 0 |
| GLDM Large Dependence Low Gray Level Emphasis | 8 | 98 | 56 | 100 | 0 | 0 |
| GLDM Low Gray Level Emphasis | 96 | 2 | 20 | 100 | 0 | 0 |
| GLDM Small Dependence Emphasis | 90 | 40 | 0 | 34 | 0 | 0 |
| GLDM Small Dependence High Gray Level Emphasis | 66 | 98 | 100 | 36 | 0 | 0 |
| GLDM Small Dependence Low Gray Level Emphasis | 100 | 12 | 34 | 12 | 0 | 0 |
| GLRLM Gray Level Non-uniformity | 100 | 76 | 100 | 28 | 100 | 0 |
| GLRLM Gray Level Non-uniformity Normalized | 62 | 4 | 58 | 52 | 0 | 0 |
| GLRLM Gray Level Variance | 42 | 74 | 8 | 80 | 0 | 0 |
| GLRLM High Gray Level Run Emphasis | 90 | 0 | 14 | 0 | 0 | 0 |
| GLRLM Long Run Emphasis | 100 | 100 | 100 | 100 | 0 | 0 |
| GLRLM Long Run High Gray Level Emphasis | 20 | 94 | 0 | 0 | 0 | 0 |
| GLRLM Long Run Low Gray Level Emphasis | 94 | 2 | 18 | 100 | 0 | 0 |
| GLRLM Low Gray Level Run Emphasis | 96 | 0 | 20 | 100 | 0 | 0 |
| GLRLM Run Entropy | 78 | 84 | 0 | 4 | 0 | 0 |
| GLRLM Run Length Non-uniformity | 100 | 100 | 90 | 0 | 0 | 0 |
| GLRLM Run Length Non-uniformity Normalized | 100 | 100 | 64 | 58 | 0 | 0 |
| GLRLM Run Percentage | 100 | 100 | 100 | 74 | 0 | 0 |
| GLRLM Run Variance | 100 | 100 | 100 | 100 | 0 | 0 |
| GLRLM Short Run Emphasis | 100 | 100 | 86 | 72 | 0 | 0 |
| GLRLM Short Run High Gray Level Emphasis | 96 | 0 | 52 | 0 | 0 | 0 |
| GLRLM Short Run Low Gray Level Emphasis | 96 | 0 | 20 | 100 | 0 | 0 |
| GLSZM Gray Level Non-uniformity | 100 | 100 | 36 | 10 | 0 | 0 |
| GLSZM Gray Level Non-uniformity Normalized | 98 | 52 | 24 | 30 | 0 | 0 |
| GLSZM Gray Level Variance | 0 | 74 | 0 | 90 | 0 | 0 |
| GLSZM High Gray Level Zone Emphasis | 100 | 0 | 72 | 0 | 0 | 0 |
| GLSZM Large Area Emphasis | 100 | 100 | 100 | 100 | 100 | 0 |
| GLSZM Large Area High Gray Level Emphasis | 100 | 100 | 100 | 100 | 100 | 0 |
| GLSZM Large Area Low Gray Level Emphasis | 100 | 100 | 100 | 100 | 100 | 0 |
| GLSZM Low Gray Level Zone Emphasis | 100 | 0 | 62 | 98 | 0 | 0 |
| GLSZM Size Zone Non-uniformity | 100 | 100 | 18 | 0 | 0 | 0 |
| GLSZM Size Zone Non-uniformity Normalized | 84 | 4 | 10 | 30 | 0 | 0 |
| GLSZM Small Area Emphasis | 84 | 4 | 8 | 30 | 0 | 0 |
| GLSZM Small Area High Gray Level Emphasis | 70 | 0 | 64 | 0 | 0 | 0 |
| GLSZM Small Area Low Gray Level Emphasis | 100 | 0 | 54 | 78 | 0 | 0 |
| GLSZM Zone Entropy | 100 | 14 | 34 | 0 | 0 | 0 |
| GLSZM Zone Percentage | 94 | 18 | 0 | 34 | 0 | 0 |
| GLSZM Zone Variance | 100 | 100 | 100 | 100 | 100 | 0 |
| NGTDM Busyness | 12 | 94 | 92 | 100 | 0 | 0 |
| NGTDM Coarseness | 100 | 100 | 70 | 38 | 0 | 0 |
| NGTDM Complexity | 100 | 74 | 100 | 64 | 0 | 0 |
| NGTDM Contrast | 100 | 98 | 98 | 82 | 0 | 0 |
| NGTDM Strength | 100 | 100 | 100 | 50 | 0 | 0 |

**Figure S2. Calibration and decision curve analysis for the three harmonization models.** The curves shown are calibrated via isotonic regression. The curves are averaged over 50 trials and the shaded regions indicate the 95% confidence interval. Brier scores (BS) for the calibration curves (top) are displayed in the upper left. For decision curves (bottom), note that the “treat none” strategy was used for these test samples when they were encountered in the clinic.

**Table S3.** **Frequency of selection for acquisition-independent radiomic features in training sets with 100% augmentation.** Features not selected after any harmonization method (0% frequency) are not shown. Features that remained acquisition-dependent after a given harmonization method, therefore not seen by the SVM, are marked by X. Frequencies are calculated over all features and trials (n = 107 features × 50 trials = 5350).

|  | **Collective Harmonization (%)** | **Covariate Harmonization (%)** | **Separate Harmonization (%)** |
| --- | --- | --- | --- |
| Shape Elongation | 66 | 50 | 6 |
| Shape Flatness | 100 | 94 | 98 |
| Shape Least Axis Length | X | 48 | 10 |
| Shape Maximum 2D Diameter Column | X | 34 | 0 |
| Shape Maximum 2D Diameter Row | X | 48 | 94 |
| Shape Maximum 2D Diameter Slice | X | 52 | 0 |
| Shape Minor Axis Length | X | 60 | 0 |
| Shape Sphericity | X | 98 | 100 |
| Shape Surface Volume Ratio | 2 | 6 | 86 |
| First Order 10 Percentile | 0 | 8 | 0 |
| First Order 90 Percentile | 38 | 12 | 0 |
| First Order Energy | X | 6 | 0 |
| First Order Entropy | 6 | X | 24 |
| First Order Interquartile Range | 14 | 28 | 52 |
| First Order Kurtosis | 44 | 70 | 98 |
| First Order Maximum | 14 | 36 | 38 |
| First Order Mean | 4 | 6 | 0 |
| First Order Range | 72 | 8 | 60 |
| First Order Robust Mean Absolute Deviation | 8 | 40 | 0 |
| First Order Root Mean Squared | 0 | 20 | 2 |
| First Order Skewness | 2 | 4 | 0 |
| First Order Total Energy | X | 6 | 0 |
| First Order Uniformity | 10 | 2 | 0 |
| First Order Variance | X | 10 | 0 |
| GLCM Autocorrelation | 0 | 10 | 0 |
| GLCM Cluster Prominence | 48 | 4 | 0 |
| GLCM Cluster Shade | 70 | 98 | 100 |
| GLCM Cluster Tendency | 36 | 12 | 2 |
| GLCM Contrast | 16 | X | 6 |
| GLCM Correlation | X | 16 | 20 |
| GLCM Difference Average | 18 | 28 | 18 |
| GLCM Difference Entropy | X | X | 16 |
| GLCM Difference Variance | 32 | 4 | 14 |
| GLCM Inverse Difference | 2 | 24 | 0 |
| GLCM Inverse Difference Moment | X | 2 | 0 |
| GLCM Inverse Difference Moment Normalized | 2 | 56 | 6 |
| GLCM Inverse Difference Normalized | X | 30 | 0 |
| GLCM Informational Measure of Correlation 1 | X | 8 | 0 |
| GLCM Informational Measure of Correlation 2 | 2 | 82 | 100 |
| GLCM Inverse Variance | 12 | 4 | 60 |
| GLCM Joint Average | 4 | 0 | 0 |
| GLCM Joint Energy | X | 18 | 10 |
| GLCM Maximum Correlation Correlation | 0 | 0 | 18 |
| GLCM Maximum Probability | X | 6 | 6 |
| GLCM Sum Average | 2 | 0 | 0 |
| GLCM Sum Entropy | 4 | 60 | 10 |
| GLCM Sum Squares | 4 | 2 | 0 |
| GLDM Dependence Entropy | X | 60 | 0 |
| GLDM Dependence Non-uniformity | X | 6 | 0 |
| GLDM Dependence Non-uniformity Normalized | 2 | 60 | 6 |
| GLDM Dependence Variance | X | X | 2 |
| GLDM Gray Level Variance | X | 4 | 0 |
| GLDM High Gray Level Emphasis | 12 | 4 | 0 |
| GLDM Large Dependence High Gray Level Emphasis | X | X | 100 |
| GLDM Large Dependence Low Gray Level Emphasis | X | X | 86 |
| GLDM Small Dependence Emphasis | 6 | 10 | 0 |
| GLDM Small Dependence High Gray Level Emphasis | X | X | 28 |
| GLDM Small Dependence Low Gray Level Emphasis | X | 38 | 0 |
| GLRLM Gray Level Non-uniformity Normalized | 22 | X | 0 |
| GLRLM High Gray Level Run Emphasis | 0 | 30 | 0 |
| GLRLM Long Run High Gray Level Emphasis | 6 | 100 | 50 |
| GLRLM Long Run Low Gray Level Emphasis | 2 | X | 0 |
| GLRLM Run Entropy | 12 | 4 | 0 |
| GLRLM Run Length Non-uniformity | X | 4 | 0 |
| GLRLM Run Length Non-uniformity Normalized | X | 8 | 0 |
| GLRLM Short Run High Gray Level Emphasis | 2 | 14 | 0 |
| GLSZM Gray Level Non-uniformity | X | 8 | 0 |
| GLSZM Gray Level Non-uniformity Normalized | 2 | 30 | 34 |
| GLSZM Gray Level Variance | 26 | 8 | 28 |
| GLSZM High Gray Level Zone Emphasis | 0 | 2 | 0 |
| GLSZM Low Gray Level Zone Emphasis | 0 | X | 6 |
| GLSZM Size Zone Non-uniformity | X | 8 | 0 |
| GLSZM Size Zone Non-uniformity Normalized | 12 | 18 | 68 |
| GLSZM Small Area Emphasis | X | 30 | 62 |
| GLSZM Small Area High Gray Level Emphasis | 28 | 36 | 0 |
| GLSZM Small Area Low Gray Level Emphasis | 0 | 10 | 100 |
| NGTDM Busyness | 4 | X | 0 |
| NGTDM Coarseness | X | 4 | 0 |
| NGTDM Complexity | X | X | 4 |
| NGTDM Contrast | X | X | 16 |
| NGTDM Strength | X | X | 100 |

**Table S4. Pearson correlation coefficients demonstrate feature selection stability across trials.** Pearson correlation was calculated for Boolean arrays indicating feature selection (1=selected, 0=not selected), determined by the L1-regularized SVM, per pair of trials for features which were acquisition-independent in all trials, the number of which varied per harmonization method. Magnitudes of the Pearson Correlation Coefficient (PCC) near 1 indicate the model gives similar importance to features across paired trials. The correlation was calculated once per nonidentical trial pairing. The numbers of feature pairings were, collective harmonization: n=136, covariate harmonization: n=1225, separate harmonization: n=1225. Collective harmonization has a different number of pairings because the test frequently failed when the input coefficient array(s) were constant due to collective harmonization’s small number of compared features. Correlation of radiomic feature values within a trial was also assessed using the PCC over n=50 trials. Features were considered correlated when PCC > 0.7. Correlations between radiomics feature values themselves may lower stability scores, as models may select different features across trials to access the same information.

|  | **Collective Harmonization** | **Covariate Harmonization** | **Separate Harmonization** |
| --- | --- | --- | --- |
| Selection PCC Mean | 1.0 | 0.48 | 0.69 |
| Selection PCC Median | 1.0 | 0.53 | 0.69 |
| Selection PCC Standard Deviation | 0.0 | 0.27 | 0.09 |
| Average Number of Correlated Feature Pairs | 0.04  [0.0-0.14] | 2.2  [1.8-2.6] | 518.5  [512.2-524.9] |
| Trials with at least one correlated feature | 2 | 50 | 50 |
| Acquisition-independent features | 2 | 10 | 100 |

**Figure S3. Displacement of samples in feature space produced by harmonization.** All unharmonized samples and harmonized samples for all trials were Z-score normalized in a common feature space. Sample displacements were then calculated in the space of features which were acquisition-independent for each trial. Displacements are reported in units of standard deviation.

**Figure S4. UMAPs of a training set before and after harmonization for a representative training set.** All training samples, unharmonized and all harmonization versions, are projected into a common two-dimensional feature space. Only features which are acquisition-independent after harmonization for the respective harmonization method are displayed in each row. Because different features are acquisition independent for different methods, the unharmonized samples are displayed as shifted in the common feature space prior to harmonization.

**Table S5. The contents of the early-development dataset.** PNs within this dataset, classified primarily by physician time-of-scan diagnosis, may be further classified by their endpoint diagnosis and endpoint diagnosis method. See Methods for a description of how endpoint diagnosis is determined and for the stability criteria used in this study for classifying some PNs.

| **Endpoint Diagnosis** | **Endpoint Diagnosis Method** | **Number of Patients** | **Number of PNs** | **Number of Scans** |
| --- | --- | --- | --- | --- |
| Malignant | PET or biopsy confirmation | 60 | 61 | 220 |
| Benign | PET or biopsy confirmation | 25 | 35 | 56 |
| Benign | Lung-RADs and stability criteria | 15 | 24 | 55 |

**Table S6. Performance metrics for the three harmonization models evaluated on only test scans with confirmed labels.** Results are reported at the scan level. Presumed-benign PNs are excluded. 100% augmentation data are included. Weighted accuracy uses balanced weighting by class (endpoint benign or malignant diagnosis). *Significant (Wilcoxon, adjusted p<0.05) improvement over collective harmonization. Due to small sample size, only improvement to PR-AUC was significant.

|  | **Collective Harmonization** | | **Covariate Harmonization** | | **Separate Harmonization** | |
| --- | --- | --- | --- | --- | --- | --- |
|  | **Mean, %** | **95% CI** | **Mean, %** | **95% CI** | **Mean, %** | **95% CI** |
| ROC AUC | 62.5 | [54.2, 70.8] | 75.2 | [66.9, 83.6] | 71.3 | [62.8, 79.8] |
| PR AUC | 73.4 | [65.8, 81.0] | 82.2* | [75.8, 88.6] | 78.3* | [71.1, 85.6] |
| Weighted accuracy | 61.5 | [56.4, 66.6] | 62 | [56.1, 67.9] | 58.1 | [51.8, 64.4] |
| Specificity at 90% sensitivity | 30.7 | [19.5, 41.9] | 49 | [36.6, 61.5] | 45.6 | [33.3, 58.0] |
| Sensitivity at 30% specificity | 79.9 | [71.4, 88.4] | 85.2 | [76.5, 94.0] | 82.4 | [73.4, 91.4] |
| Sensitivity at 90% specificity | 35.1 | [23.7, 46.5] | 51.3 | [39.1, 63.4] | 44.9 | [31.8, 57.9] |

|  | **R^2^, +log**  **Collective Harmonization** | | **R^2^, +log**  **Covariate Harmonization** | | **R^2^, +log**  **Separate Harmonization** | |
| --- | --- | --- | --- | --- | --- | --- |
| **Train : Test** | **ROC-AUC** | **PR-AUC** | **ROC-AUC** | **PR-AUC** | **ROC-AUC** | **PR-AUC** |
| CE : Non-CE | - | - | - | - | - | - |
| Non-CE : CE | 0.31 | 0.18 | 0.18 | 0.04 | 0.91 | 0.86 |
| GE : SIEMENS | 0.83 | 0.39 | 0.75 | 0.63 | 0.83 | 0.77 |
| SIEMENS : GE | 0.79 | 0.76 | 0.82 | 0.79 | 0.87 | **-** |

**Figure S5. Test cases for pipeline performance on unseen batch effects.** Performance is calculated at the scan level. The learning curves for models trained on early-development scans acquired with one manufacturer or CE status and tested on the held-out set, obtained by varying the fraction of available augmentation data included in the training set. Multiple trials for 5%-75% augmentation were obtained by varying the random splitting of the available augmentation data into augmentation fractions. 20 trials were attempted for each point, but some trials failed at low augmentation due to rare acquisition parameters. Error bars show the 95% confidence interval. Per curve, the data is not harmonized for the acquisition parameter by which the split is made, and only augmentation data corresponding to the training batch is included, creating an unseen batch effect. The leftmost point in each plot represents the baseline un-augmented model. Higher baseline performance compared to our primary pipeline is likely due to removing, necessarily, the constraint isolating scans from a patient within a fold. Performance increases with augmentation fraction for three of four test cases. Data from CE scans does not generalize well to non-CE scans. Gains from augmentation were not stable for PR-AUC when training on separately harmonized Siemens data and testing on GE. Where data could be fitted to a positive logarithmic curve,$R^{2}$ fits are given in the associated table.

**Table S7. Acquisition parameter-based training scan exclusion.** Mean numbers of training scans excluded per trial due to rare instances of an acquisition parameter. All excluded scans were later-development scans. Rare focal spots was responsible for 35% of exclusions, rate KVP was responsible for 33% of exclusions, rare CE (during separate harmonization) was responsible for 24% of exclusions, and rare manufacturer was responsible for 8% of exclusions. Trial numbers *n*, from top to bottom—collective harmonization: *n*=50 for all, covariate harmonization: *n*=50 for all, separate harmonization: *n*=[50, 10, 30, 45, 50, 50, 50].

|  | **Collective Harmonization** | | **Covariate Harmonization** | | **Separate Harmonization** | |
| --- | --- | --- | --- | --- | --- | --- |
| **Augmentation fraction (%)** | **Mean** | **95% CI** | **Mean** | **95% CI** | **Mean** | **95% CI** |
| 0 | 0 | [0 - 0.07] | - | - | - | - |
| 5 | 0.1 | [0.03 - 0.23] | 0.1 | [0.03 - 0.23] | - | - |
| 10 | 0.1 | [0.03 - 0.23] | 0.1 | [0.03 - 0.23] | - | - |
| 15 | 0.1 | [0.03 - 0.23] | 0.1 | [0.03 - 0.23] | 7.5 | [5.90 - 9.40] |
| 20 | 0.1 | [0.03 - 0.23] | 0.1 | [0.03 - 0.23] | 5.17 | [4.39 - 6.05] |
| 25 | 0.3 | [0.17 - 0.49] | 0.3 | [0.17 - 0.49] | 4.67 | [4.06 - 5.34] |
| 50 | 0.7 | [0.49 - 0.97] | 0.7 | [0.49 - 0.97] | 3.9 | [3.37 - 4.49] |
| 75 | 0.9 | [0.66 - 1.20] | 0.9 | [0.66 - 1.20] | 1.9 | [1.54 - 2.32] |
| 100 | 1 | [0.74 - 1.32] | 1 | [0.74 - 1.32] | 2 | [1.63 - 2.43] |

**Figure S6.** **The average performance of the three harmonization models with hyperparameters tuned via nested cross-validation.** a. (c.) The ROC-AUC (PR-AUC) learning curves for hyperparameter-tuned models with augmented training sets obtained by varying the fraction of augmentation data included in the training set. The leftmost point in each plot represents the baseline un-augmented model. Error bars show the 95% confidence interval over trials where a model could be trained. Trial numbers *n* for each point, from left to right—collective harmonization: *n*=50 for all, covariate harmonization: *n*=50 for all, separate harmonization: *n*=[50, 10, 30, 45, 50, 50, 50]. Separate harmonization failed for 5% and 10% augmentation due to small sample size, so these points are omitted. b. (d.) Receiver operating (precision-recall) curves for hyperparameter-tuned models trained using 100% of augmentation data, averaged over all trials, n=50 for each. Shaded regions show the 95% confidence interval. Gray lines indicate chance-level performance.
